# Designing Synergistic Biostimulants Formulation Containing Autochthonous Phosphate-Solubilizing Bacteria for Sustainable Wheat Production

**DOI:** 10.3389/fmicb.2022.889073

**Published:** 2022-05-03

**Authors:** Mahreen Yahya, Maria Rasul, Yasra Sarwar, Muhammad Suleman, Mohsin Tariq, Syed Zajif Hussain, Zahid Iqbal Sajid, Asma Imran, Imran Amin, Thomas Reitz, Mika Tapio Tarkka, Sumera Yasmin

**Affiliations:** ^1^Soil and Environmental Biotechnology Division, National Institute for Biotechnology and Genetic Engineering College, Pakistan Institute of Engineering and Applied Sciences (NIBGE-C, PIEAS), Faisalabad, Pakistan; ^2^Department of Environment and Energy, Sejong University, Seoul, South Korea; ^3^Health Biotechnology Division, National Institute for Biotechnology and Genetic Engineering College, Pakistan Institute of Engineering and Applied Sciences (NIBGE-C, PIEAS), Faisalabad, Pakistan; ^4^School of Life Sciences, Institute of Microbiology, Lanzhou University, Lanzhou, China; ^5^Department of Bioinformatics and Biotechnology, Government College University Faisalabad (GCUF), Faisalabad, Pakistan; ^6^Department of Chemistry and Chemical Engineering, SBA School of Science and Engineering (SBA-SSE), Lahore University of Management Sciences (LUMS), Lahore, Pakistan; ^7^Agricultural Biotechnology Division, National Institute for Biotechnology and Genetic Engineering College, Pakistan Institute of Engineering and Applied Sciences (NIBGE-C, PIEAS), Faisalabad, Pakistan; ^8^Soil Ecology Department, UFZ-Helmholtz-Centre for Environmental Research, Halle, Germany; ^9^German Centre for Integrative Biodiversity Research (iDiv) Halle-Jena-Leipzig, Leipzig, Germany

**Keywords:** marker genes, *gcd*, *pqqC*, *pho*, *Ochrobactrum*, FESEM, bioformulations, FISH

## Abstract

Applying phosphate-solubilizing bacteria (PSB) as biofertilizers has enormous potential for sustainable agriculture. Despite this, there is still a lack of information regarding the expression of key genes related to phosphate-solubilization (PS) and efficient formulation strategies. In this study, we investigated rock PS by *Ochrobactrum* sp. SSR (DSM 109610) by relating it to bacterial gene expression and searching for an efficient formulation. The quantitative PCR (qPCR) primers were designed for PS marker genes glucose dehydrogenase (*gcd*), pyrroloquinoline quinone biosynthesis protein C (*pqqC*), and phosphatase (*pho*). The SSR-inoculated soil supplemented with rock phosphate (RP) showed a 6-fold higher expression of *pqqC* and *pho* compared to inoculated soil without RP. Additionally, an increase in plant phosphorous (P) (2%), available soil P (4.7%), and alkaline phosphatase (6%) activity was observed in PSB-inoculated plants supplemented with RP. The root architecture improved by SSR, with higher root length, diameter, and volume. *Ochrobactrum* sp. SSR was further used to design bioformulations with two well-characterized PS, *Enterobacter* spp. DSM 109592 and DSM 109593, using the four organic amendments, biochar, compost, filter mud (FM), and humic acid. All four carrier materials maintained adequate survival and inoculum shelf life of the bacterium, as indicated by the field emission scanning electron microscopy analysis. The FM-based bioformulation was most efficacious and enhanced not only wheat grain yield (4–9%) but also seed P (9%). Moreover, FM-based bioformulation enhanced soil available P (8.5–11%) and phosphatase activity (4–5%). Positive correlations were observed between the PSB solubilization in the presence of different insoluble P sources, and soil available P, soil phosphatase activity, seed P content, and grain yield of the field grown inoculated wheat variety Faisalabad-2008, when di-ammonium phosphate fertilizer application was reduced by 20%. This study reports for the first time the marker gene expression of an inoculated PSB strain and provides a valuable groundwork to design field scale formulations that can maintain inoculum dynamics and increase its shelf life. This may constitute a step-change in the sustainable cultivation of wheat under the P-deficient soil conditions.

## Introduction

Phosphorus (P) is an essential key macro-nutrient for the optimal plant growth (Brito et al., 2020; Chandran et al., 2021) and a crucial element for various physiological and biochemical functions at plant cellular level (Bechtaoui et al., 2021). The complex dynamics of P in soil makes it a limited resource for plant uptake (Bindraban et al., 2020). To satisfy the rising P nutrition requirements of crops and to boost agricultural production, the conventional agriculture relies on agrochemicals, a practice that compromises the human and environmental health (Akanmu et al., 2021). Further, the global P input into croplands is expected to increase by up to 86% by 2050 (Mogollón et al., 2018), and thus sustainable means for P fertilization are urgently needed.

The application of bacteria that are beneficial for crop production, commonly termed as plant growth-promoting bacteria, has been adopted as a potent, biological alternative for the mineral fertilization. The bacterial inoculants can be used as biofertilizers and biostimulants to improve the soil nutrient availability, plant nutrient uptake and assimilation, and root growth, since their biological activities include, e.g., mineral and organic phosphate solubilization (PS), nitrogen fixation, siderophore, and indole-3-acetic acid production (Castiglione et al., 2021). Among the plant beneficial bacteria, the biofertilizer group phosphate-solubilizing bacteria (PSB) are promising candidates to satisfy the P requirements of the plants by converting unavailable soil inorganic (P_i_) and organic (P_o_) forms of phosphate into plant-available orthophosphates through dissolution and absorption (Chen and Liu, 2019). The P_i_-solubilizing microorganisms secrete organic acids to dissolve phosphate minerals, whereas the organic phosphates are hydrolyzed by acid and alkaline phosphatases as well as phytases (Liu et al., 2020). In general, the application of PSB in soil decreases the pH locally and consequently makes the P bound in minerals available to the plant and strengthens the activity of other beneficial microorganisms (Etesami et al., 2021).

At present, the research on the genes of PSB related to P_i_ solubilization is mainly focused on *pyrroloquinoline quinone–glucose dehydrogenase (gcd)* and six redox–coenzyme *pyrroloquinoline quinone* genes *pqq A, B, C, D, E, F*, and *G* (Bhanja et al., 2021). By contrast, the organic phosphates are hydrolyzed by acid phosphatases, alkaline phosphatases, and phytases (inositol phosphate phosphatases) that are encoded by *phosphatase* (*pho*) genes (Sarr et al., 2020). The knowledge on the genetic potential of PSB is still scanty, and the molecular studies to understand how PSB brings out the P solubilization are inconclusive (Liu et al., 2018).

In spite to the wide varieties of PSB, i.e., *Acinetobacter, Bacillus, Enterobacter Ochrobactrum*, and *Pseudomonas*, there are still relatively limited studies on P-solubilizing pathways and expression patterns of genes related to phosphate-solubilizing that need to be further studied. For this reason, the first aim of this work was to construct primers and to provide expression analysis of key PS related genes, i.e., *gcd, pqqC*, and *pho* of selected PSB.

Although an elite strain with a high and consistent PS activity is an essential prerequisite for the development of successful inoculant, the non-biological components for bioformulations are still key bottlenecks in commercial development of the inoculants (Mendoza-Suárez et al., 2021). The choice of carrier material is fundamental, because carrier is the sole delivery vehicle of live microorganism from the production unit to the plants in the field (Vassilev et al., 2020). The main roles of a good carrier material are as follows: (i) To provide an optimal microenvironment for microorganisms to maintain microbial viability and longer shelf life without any need for special storage and (ii) to support the strain(s) competitive abilities with the usually much better adapted, native soil microflora (Soumare et al., 2020). Besides, the carrier material must be readily available, easily pulverized and sterilized, cost-effective, compatible with the environment, acquiescent to nutrient supplement, and un-harmful to the user (Koskey et al., 2021). The carrier materials can be organic [e.g., compost (CM), biochar (BC), peat, biogas, humic acid (HA), slurry, etc.] or inorganic (e.g., talc, perlite, lignin, zeolite, etc.) origin, or even synthesized from specific compounds (Mitter et al., 2021). The by-products from the food and agricultural industries are also important sources for the development of biostimulants. The PSB-based carrier materials can include extracts from food waste, manures, vermicomposting, aquaculture waste streams or even sewage treatments (Madende and Hayes, 2020).

Despite the great potential and long-term expedient effects, the biostimulants/biofertilizers still face major challenges that limit their use in agriculture. These challenges are often associated with limited shelf life and survival of inoculated strain in the field, in variable environments and with different host plants (Mitter et al., 2021). For this reason, the various commercial bioinoculants did not function under field conditions with a similar efficacy as in greenhouse or laboratory conditions (Stamenkovi et al., 2018; Santos et al., 2019; Vassilev et al., 2020). Mostly, the reasons for that were inadequate formulations with carriers that did not support the survival and growth of the microorganisms and/or were not stable under field conditions (Fasusi et al., 2021). Furthermore, the simultaneous application of two or more plant growth promoting rhizobacteria (PGPR) as consortium can improve the plant growth and can cause substantial increment in the crop yield (Shahzad et al., 2017; Backer, 2018; Mpanga et al., 2019). Bacteria in the consortium can efficiently colonize in rhizosphere than single bacterium (Molina-Romero et al., 2021) and they not only reduce the application chemical fertilizers but also allow the plants to utilize P already present in the soil (Lobo et al., 2019). Hence, the second aim of this study was to design a successful bioformulation based on PSB consortium with optimal shelf life and agro-industrial by-products as sole carbon source/organic amendments. We expected that this approach is expedient for developing a robust and effective alternative for mineral P fertilization.

We hypothesized that (H1) the autochthonous PSB that can solubilize different unavailable forms of phosphate, may have the genetic potential to express key genes responsible for PS and improve root architecture concomitantly under P-deficient conditions. We assumed (H2) that designing biostimulants based on a PSB consortium and using different agro-industrial by-products as organic amendments could result in a product with a long shelf life and survival in the wheat rhizosphere. We also expected (H3) correlations between P solubilization from the different insoluble phosphate sources by PSB and increased soil available P, consequently also resulting in improved wheat root growth, and grain yield.

## Materials and Methods

### The PSB and Evaluation of Their P-Solubilization Potential in Different Insoluble P Sources

The PSB *Enterobacter* spp. ZW9, ZW32 (GenBank accession numbers MK422617 and MK817561) and *Ochrobactrum* sp. SSR (GenBank accession number MK422612) used in this study were isolated from the wheat rhizosphere and characterized in our previous study (Yahya et al., 2021). These PSB are non-pathogenic, compatible with each other and were also submitted to DSMZ (https://www.dsmz.de/) culture collection under the accession numbers DSM 109610 and DSM 109592, DSM 109593 for *Ochrobactrum* sp. SSR, *Enterobacter* sp. ZW9 and *Enterobacter* sp. ZW32, respectively.

In the current study, an *in vitro* assay was carried out to assess the P-solubilization potential of P-solubilizing bacteria SSR, ZW32 and ZW9 in the presence of different insoluble P sources. The three PSB strains were cultured in rock phosphate (RP) broth medium (Nguyen et al., 1992) and phytate broth medium (Howson and Davis, 1983) supplemented with insoluble P sources, i.e., RP and phytate, respectively. The flasks were kept at 28 ± 2°C and 180 rpm. An un-inoculated medium was used as a control. The P-solubilizing abilities of PSB were quantitatively estimated at 3, 5, 7, 10, and 15 days post-inoculation (DPI). To get the cell-free supernatant, the cultures were centrifuged at 4,000 rpm for 10 min at 4°C. The supernatant obtained was used for available P quantification as described by Murphy and Riley (1962) using the molybdenum blue method.

### Expression Analysis of Genes Responsible for P-Solubilization in Soil Amended With RP Using Quantitative Real-Time PCR (qRT-PCR)

Based on the *in vitro* P-solubilizing efficacy and genetic potential (Table 1), *Ochrobactrum* sp. SSR was selected for expression analysis of PS-related genes as compared to the other two potential PSB (*Enterobacter* spp. ZW32 and ZW9). Therefore, a pot experiment was carried out in the soil amended with RP to evaluate the effect of SSR inoculation on wheat growth and root architecture concomitantly with expression of key PS related genes.

**Table 1:** The qPCR primers for quantification of genes encoding for proteins involved in PS, i.e., *gcd, pqqC*, and *pho*.

**Bacterial strain**	**Primer name**	**Primer sequence**	**Product length (bp)**	**Gene length (bp)**	**Reference**
*Ochrobactrum* sp. SSR	F26_pqqC_F	GGGCTTGACCCCGACTATGT	360	783	This study
	R26_pqqC_R	CGACGGCATCCTGCTTTTCC			
	F_pqqE_F	TTYTAYACCAACCTGATCACSTC	725	-	Perez et al., 2007
	R_pqqE_F	TBAGCATRAASGCCTGRCG			
	F12_gcd_F	TCACGACCTCTGGGACTACG	847	2439	Rasul et al., 2021
	R12_gcd_R	CTTCCACAATTCCCGACCCG			
	P12_gcd_F	ATGTCACGGGCGTCGATCTG	131	265	This study
	P12_gcd_R	CGTGATCATCGGTCCGCCTA			
	P26_pqqC_F	GCTTTGCGGTGGGTGCTT	78	222	This study
	P26_pqqC_R	GTTCTGTCAGCGACGATGCG			
	P30_phosphatase_F	ATGCGCGACCGAAAGACC	19	191	Rasul et al., 2021
	P30_phosphatase_R	TGCTGGCGACGAGACGAA			

### Effects of *Ochrobactrum* sp. SSR Inoculation on Wheat Plants and Soil Amended With RP

The PSB *Ochrobactrum* sp. SSR was evaluated for phosphate-solubilizing potential in soil amended with insoluble RP under the greenhouse conditions at National Institute for Biotechnology and Genetic Engineering (NIBGE), Faisalabad (31°25′0″N 73°5′28″E). Seeds of the wheat variety Faisalabad-2008 were surface sterilized with sodium hypochlorite (1.5%) and sequentially washed with sterile water. For the inoculum preparation, a single colony of SSR was grown in Luria–Bertani (LB) broth medium at 28 ± 2°C for 24–48 h. Sterilized seeds were inoculated with SSR (1 × 10^9^ CFU ml^−1^) for 30 min. Un-inoculated seeds dipped in LB medium were used as control. Two seeds per treatment were sown in cylindrical pots (10-cm diameter) filled with 300-g soil (loam texture, 0.57 % organic matter, pH 8, and 2 mg kg^−1^ available P) per pot.

The four biological treatments, i.e., T_1_: SSR-inoculated seeds and soil amended with 45-mg P in the form of RP; T_2_: Un-inoculated seeds and soil supplemented with 45-mg P in the form of RP; T_3_: SSR-inoculated seeds and soil without RP; T_4_: Un-inoculated seeds and soil without RP, each with six biological replicates, were arranged in a completely randomized design. All pots were provided with basic nutrient solution (Hoagland and Arnon, 1950) before sowing. Then T1 and T2 were provided with Hoagland without any P source while, T3 and T4 received Hoagland solution with complete nutrients. After germination, two plants per pot were grown and pots were watered as per requirement.

The plants were uprooted 30 days after sowing (DAS) and the plant growth parameters, i.e., root length, shoot length, plant dry, and fresh weight were determined. The root morphological parameters, i.e., root length (cm), root surface area (cm^2^), root volume (cm^3^), root length per volume (cm m^−3^), root diameter (cm), number of crossings, number of forks, and tips were measured by using a rhizoscanner (Epson photo scanner V-700, USA) accompanied with WinRHIZO software (Regent Int. Co., Ltd. Canada).

Plant P was analyzed by the tri-acid digestion method according to Tandon (1993). Rhizosphere soil was analyzed for available P by the Olsen method (Olsen, 1954) and alkaline phosphatase activity by following the *p*-nitrophenyl method (Tabatabai and Bremner, 1969).

### Quantitative Real Time PCR (qRT-PCR)

To validate the persistence of inoculated PSB, wheat rhizosphere soil was collected from inoculated treatments of the pot experiment at 30 DAS and qRT-PCR was performed for expression analysis of PSB genes. Strain-specific (*Ochrobactrum* sp. SSR) primers for genes, i.e., glucose dehydrogenase (*gcd*), pyrroloquinoline quinone biosynthesis protein C gene *pqqC* and phosphatase (*pho*) responsible for P solubilization were used for expression analysis.

#### Primers Designing and Phylogenetic Analysis of PS-Related Genes

The primers for the amplification of *pqqC* genes of *Ochrobactrum* sp. SSR, encoding proteins involved in inorganic P solubilization were designed according to Rasul et al. (2021). The primers for *gcd* and *pho* gene of *Ochrobactrum* sp. SSR have been designed and reported in our preceding study (Rasul et al., 2021). The primers used in PCR amplification to evaluate the functionality and to authenticate the predicted sizes of amplicons (Table 1). The PCR conditions were 98°C for 3 min, followed by 35 cycles of 95°C for 60 s followed by 54°C for 60 s, 72°C for 60 s and final elongation at 72°C for 10 min. The amplified PCR-products were run on agarose gel (1%) at 80 V for 45 min. The PCR products were purified using PCR purification kit (QIAGEN Science, USA) and sequenced by Macrogen (Seoul, Korea). The obtained sequences were aligned and compared to sequences available at NCBI (https://www.ncbi.nlm.nih.gov/) and deposited to GenBank. Phylogenetic analysis was carried out by using MEGA6 software by using maximum likelihood method (Kumar et al., 2016).

#### Primer Design for qRT-PCR

*Gcd* and *pqqC* primers for qRT-PCR were designed for *Ochrobactrum* sp. SSR and were used in PCR amplifications to verify the amplification of the predicted amplicon sizes (Table 1). The primers for *pho* gene were designed and reported in the previous study (Rasul et al., 2021). The *Gcd* gene was selected as reference gene to normalize and validate qRT-PCR experiment by screening relative expression (based on fold change values) of candidate genes, i.e., *pqqC* and *pho* genes.

#### The RNA Extraction and cDNA Synthesis

The RNA was extracted from the soil with FastRNA spin Kit (MP, Biomedical, USA) by following the manufacturer's instructions. The cDNA was synthesized using First-Strand cDNA Synthesis Kit (Invitrogen, California, USA). The quality and quantity of cDNA were analyzed using Nanodrop 2000 (Thermo 262 Scientific, United States).

#### Expression Analysis by qRT-PCR

Bio-Rad RT-PCR (Bio-Rad, Hercules, USA) with SYBR Premix ExTaq kit (Takara Bio, USA) were used for expression analysis. A 25-μL reaction mixture contained 12.5-μL SYBR Green Master, 0.2-μL forward primer (10 μM), 0.2-μL reverse primer (10 μM), 2-μL cDNA, and 10.1 μL deionized water. The amplification conditions were as follows: The initial denaturation at 95°C for 5 min, 35 cycles of 95°C for 30 s followed by 54°C for 30 s, and 72°C for 30 s. The melting curve was analyzed from 54°C to determine the primer specificity.

The data were calculated as described by Javaid et al. (2016) and the expression level of target gene *pqqC* and *pho* were normalized with the C_t_-value of reference gene (*gcd*). All samples were analyzed in three replicates.

### Designing of Bioformulations Based on PSB-Consortium and Organic Amendments

Four different carrier materials, i.e., BC, CM, filter mud (FM), and HA were used to design bioformulations with PSB consortium comprising of well characterized strains (*Ochrobactrum sp*. SSR, *Enterobacter* spp. ZW32, and ZW9). The BC was obtained from wheat straw by pyrolysis in an automated furnace at 350°C with a resident time of 1 h and at a constant heat rate, i.e., 10°C min^−1^ (Abbas et al., 2020). The CM was derived from cow dung together with nitrogen-rich plant material and was obtained from Microbial Ecology Lab (NIBGE, Pakistan). The FM was obtained from decantation and filtration of sugarcane waste product (Suleman et al., 2018). The HA was extracted by alkali treatment of lignite as described by Ghani et al. (2021).

The standard procedures were followed for the characterization of carrier materials. The pH of the carrier materials was estimated by using pH meter (Phs-3c, Rex, China) according to Thomas (1996). The electric conductivity of soil was determined by using electric conductivity meter (DDS307A, Rex, Shanghai; Rhoades, 1993). The soil organic matter was estimated by Walkley black wet digestion method (Nelson and Sommers, 1996). Kjeldal method was used to measure total nitrogen (Bremner and Mulvaney, 1982). Elemental analysis of N and C was further validated by using CHNS analyzer (Perkin Elmer, Massachusetts, United States). The total P was determined by vanadate-molybdate method (Olsen, 1954).

The carrier materials were ground, sieved through 2-mm sieve and autoclaved. For the development of consortium, PSB strains, i.e., *Ochrobactrum* sp. SSR, *Enterobacter* spp. ZW32, and ZW9 were grown in LB broth medium separately at 28 ± 2°C for 24–48 h. The consortium (1 × 10^9^ CFU ml^−1^) was developed by mixing equal volume of each of the bacterium that are grown in LB. The bacterial suspension was mixed uniformly and aseptically with the carrier material to allow an adequate evenness to the final mix. Each carrier was prepared aseptically by adding 300 ml of bacterial suspension to 700 g of carrier and mixed well. The negative controls were also prepared by aseptically adding 300 ml of un-inoculated LB broth to 700 g of carrier. Each bioformulation and negative controls were prepared in triplicates, stored in polythene bags and were incubated at 28°C until the sampling time (Pastor-Bueis et al., 2019).

### Shelf Life Study of Bioformulations

The survival of the inoculated PSB was assessed in all tested formulation at different time intervals, i.e., after 15, 30, 60, 90, 180, and 270 days. Three samples from each formulation were collected at each sampling date and analyzed for survival of the PSB inoculum. The bacterial viability was estimated for each sample by four-fold serial dilutions on LB agar and NBRIP (National Botanical Research Institute's Phosphate) (Nautiyal, 1999) agar supplemented with tri-calcium phosphate as source of P. The mean values of PSB viable count per gram of carrier material was calculated at each time interval and plotted on logarithmic scale (Pastor-Bueis et al., 2019).

### Morphological and Elemental Analysis of Bioformulations by Scanning Electron Microscopy With Energy Disruptive X-Ray Spectroscopy Microanalysis (SEM/EDS)

Morphology or surface features of PSB inoculated and un-inoculated bioformulations were observed using field emission scanning electron microscopy (FESEM). The predominant chemical elements were determined by element microanalysis through EDS with a JEOL SEM microscope JSM 6490-LV model at a voltage range 10–20 kV and 1,000–10,000 magnification (Shahzad et al., 2019).

### *In-vivo* Evaluation of Bioformulations in Earthen Pots Under Net House Conditions

The bioformulations were evaluated for their impact on wheat grain yield and plant P content under net house conditions at NIBGE, Faisalabad (31°23'45.1 “N, 73°01'3.4”E) in earthen pots through wheat-growing season November 2018 to April 2019. The seeds of wheat variety Faisalabad-2008 were surface sterilized as illustrated in preceding section. After sterilization, the seeds were pelleted with each bioformulation (@ 50-kg seeds kg^−1^carrier material), separately and kept for 30 min. Un-inoculated pelleted seeds (with carrier materials) were used as control.

The sowing was done in the earthen pots (30-cm diameter) filled with 12-kg soil (loamy texture, pH 8.2, soil available P 1.9 mg kg^−1^ and organic matter 0.6%) and watered prior to sowing. The soil was supplemented with 80% recommended di-ammonium phosphate (DAP) dose (i.e., 20% reduced DAP). Eight treatments, six biological replicates, and four plants per pot were arranged in two factorial completely randomized design (Supplementary Table S2). Fertilizers were applied as per the recommendations, where DAP (150-mg kg^−1^ soil) and urea (75-mg kg^−1^ soil) in split doses were supplemented at the time of sowing and with first and second watering.

After 35 days of sowing plants were uprooted and multiple plant growth parameters (root length, shoot length, and plant dry weight) were evaluated. At maturity, plants were harvested and wheat yield parameters, i.e., number of tillers, grain yield, plant biomass, and plant height were recoded. Plant P content (Tandon, 1993), available P (Olsen, 1954), and alkaline phosphatase activity of soil (Tabatabai and Bremner, 1969) were analyzed by standard protocols.

### *In planta* Evaluation of Bioformulation for Wheat Yield Parameters Under Field Conditions

#### Microplot Experiment

Based on the improved wheat yield and higher P-solubilizing potential in the net house experiment, the FM-based bioformulation was selected and evaluated in microplots at NIBGE. Each microplot was of the size of 1.5 × 1.5 m.

Three biological replicates for three treatments including seeds primed with bioformulation and soil supplemented with 80% of the recommended dose of DAP, i.e., 20% reduced dose and un-inoculated controls supplemented with 80 and 100% DAP doses, respectively, were arranged in randomized complete block design. Seed pelleting of the wheat variety, Faisalabad-2008, was carried out as described in the previous section. The seeds were sown with the help of a dibbler in microplots. Fertilizers were applied as per recommendations (N: P, 15:100-kg ha^−1^). The DAP and urea in split doses were applied at the time of sowing and subsequently with first and second irrigations.

### Field Trial

A field trial was conducted to evaluate the selected bioformulation during winter season of November 2019–April 2020 at NIBGE, Faisalabad (31°23'45.1 “N, 73°01'3.4”E).Three biological replicates for three treatments, i.e., seeds primed with selected bioformulation provided with 80% recommended dose of DAP and un-inoculated controls supplemented with 80 and 100% doses of DAP, respectively were arranged in randomized complete block design. The seeds were sown by drill method in a plots size of 6 m × 6 m. Doses of fertilizers, treatments, and controls were similar to that applied in microplot experiment. While, standard agronomic practices were maintained throughout the experiment.

### Estimation of Wheat Yield Parameters, Seed P, Available Soil P, and Phosphatase Activity

At maturity, the plants from both microplots and field trial were harvested and wheat yield parameters, i.e., number of tillers, grain yield, plant biomass, and plant height were recoded. Plant P content (Tandon, 1993), available P (Olsen, 1954), and alkaline phosphatase activity of soil (Tabatabai and Bremner, 1969) were analyzed by standard protocols.

### Detection of Inoculated PSB

The survival of inoculated PSB was evaluated through the viable count method (Somasegaran and Hoben, 2012). The persistence of inoculated PSB was confirmed by fluorescent *in situ* hybridization (FISH) equipped with confocal laser-scanning microscopy (CLSM) (Amann et al., 1995). Inoculated *Ochrobactrum* sp. SSR was detected by hybridizing the Cy3-labeled ALF1b probe. While FLUOS-labeled probe “EUB338” was used for the total bacterial population (Manz et al., 1996). An Argon–Ion laser was used for excitation of FLOUS at 488 nm and Cy3 at 514 nm. FV10-ASW 1.7 software was used for imaging (Schneider et al., 2012).

The morphological characteristics and multiple plant growth promoting traits, i.e., phosphate solubilization, production of gluconic acid, and zinc solubilization of re-isolated PSB were compared to that of inoculated PSB (Yasmin et al., 2016).

### Statistical Analysis

The data derived from *in vitro* experiments, pot experiment and field trials were statistically analyzed by ANOVA. The least significant difference (LSD) compared the variations between the treatments at 1 and 5% confidence level for lab assessments and field trials respectively, by using software STATISTIX-10 (Tallahassee, FL, USA). For the principal component analysis SPSS 23.0 software (SPSS Inc., USA) was used. Box plots were generated for analysis of wheat yield parameters and soil parameters in an earthen pot experiment by using Origin Software 2020b (Origin Lab Corporation Northampton, MA, USA).

## Results

### The PSB and Evaluation of Their P-Solubilization Potential in Different Insoluble P Sources

All tested PSB strains, i.e., *Ochrobactrum* sp. SSR, *Enterobacter* spp. ZW32, and ZW9 exhibited P-solubilizing activity in RP medium and phytate medium (Supplementary Table S1). The highest level of solubilized P from RP (112 μg ml^−1^) was detected with SSR 15 DPI. The maximum P solubilization from phytate occurred 10 DPI for ZW32 and 3 DPI for ZW9 and SSR (Supplementary Table S1). The P released from phytate by SSR at 15 DPI (22 μg ml^−1^) was considerably lower than from RP (112 μg ml^−1^) (Supplementary Table S1).

### Phylogenetic Analysis of Genes Responsible for P-Solubilization

**Figure 1:**
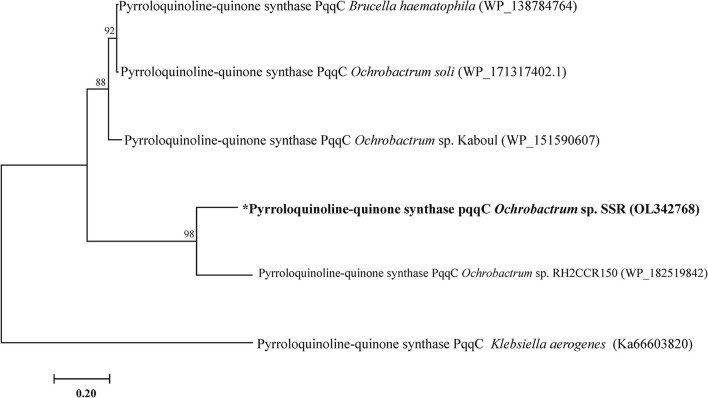
Phylogenetic tree of pqqC gene of *Ochrobactrum* sp. SSR. Phylogenetic trees was constructed by maximum likelihood method.

The PQQ biosynthesis protein C related gene *pqqC* (GenBank under accession number OL342768) was amplified from genomic DNA of SSR. It showed 98% identity to *pqqC* nucleotide sequence of *Ochrobactrum* sp. (Figure 1).

### Expression Analysis of *gcd, PqqC*, and *pho* Genes by qRT-PCR

**Figure 2:**
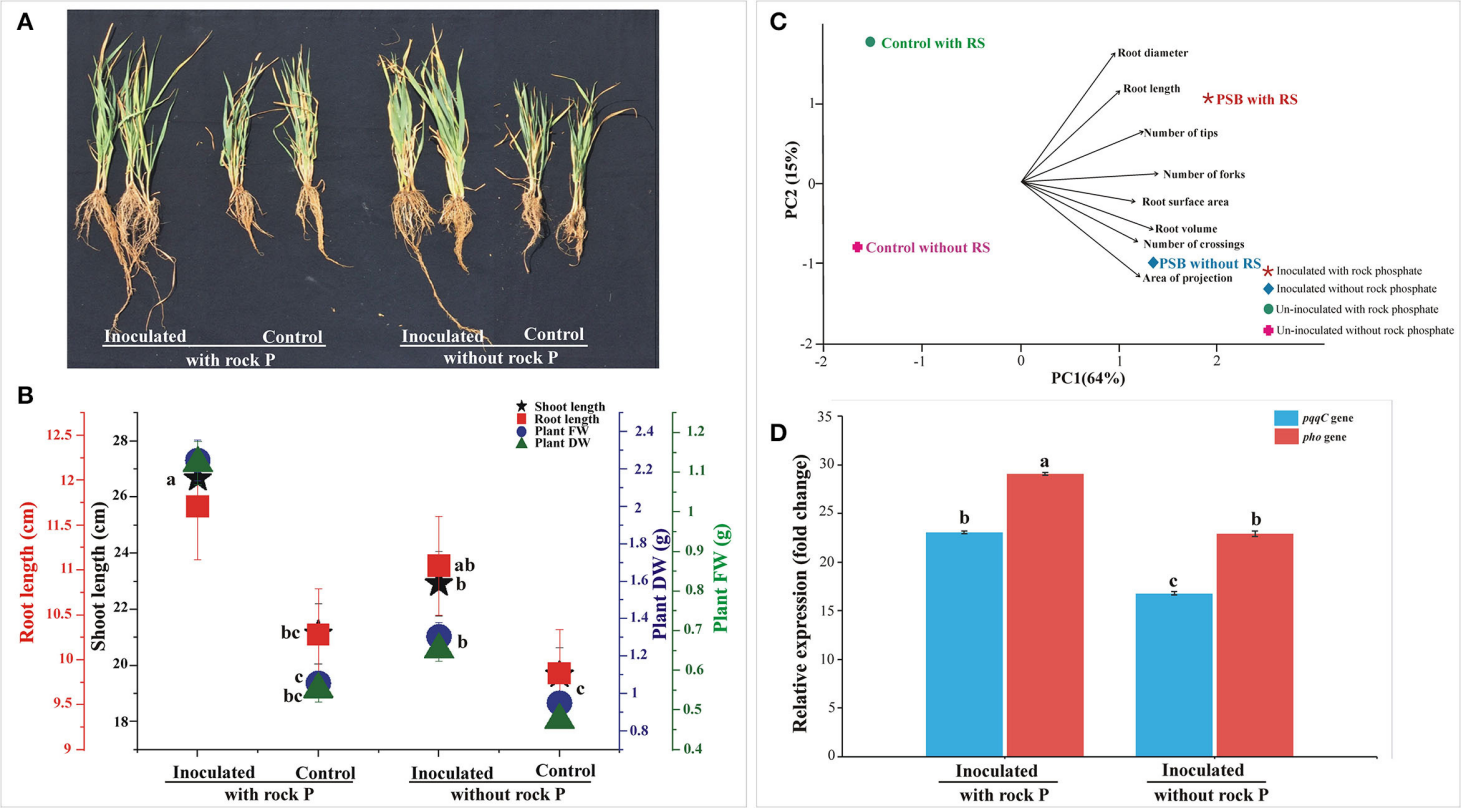
*In vivo* Screening of PSB inoculation in soil amended with RP and analysis of bacterial gene expression. The data was recorded at 30 days of cultivation. Plants were used to evaluate different plant growth parameters **(A,B)** and root parameters **(C)**. Principal component analysis linking SSR inoculation to wheat root architecture **(C)**. The components elucidate a total variance of 79%. The loadings showed strength and direction of root parameters in dataset. The relative expression profile of *pqqC* and *pho* encoding gene of *Ochrobactrum* sp. SSR, expressed in the soil of the different treatments **(D)**. The data represents the relative fold changes in the expression values of the *pqqC* and phosphatase gene of *Ochrobactrum* sp. SSR in the soil with respect to *gcd* gene. Bars with distinct alphabetical letters are significantly different at *p* ≤ 0.05.

The persistence of *Ochrobactrum* sp. SSR in wheat rhizosphere was estimated by gene expression analysis of the *gcd, pqqC*, and *pho* genes gene. Among the tested treatments, the highest abundance of SSR was observed in RP amended soil. The supplementation of the soil with RP led to increased *gcd, pqqC*, and *pho* expression level (Figure 2D).

The relative expression level of *pqqC* and *pho* gene was 23-fold and 29-fold higher than reference gene *gcd* in SSR inoculated treatment supplemented with RP, while the expression level of *pho* gene was 3-fold higher than *pqqC* gene. On the other hand, the relative expression level of *pqqC* and *pho* gene was 17-fold and 23-fold higher, respectively, than the reference gene *gcd* in SSR inoculated treatment without RP (Figure 2D), while the expression level of *pho* gene was 6-fold higher than that of *pqqC* gene. Collectively, the SSR-inoculated treatment supplemented with RP showed high expression levels for *pqqC* and *pho* gene, i.e., 6-fold higher compared to inoculated treatment without RP (Figure 2D).

### Effects of PSB Inoculation on Wheat Plants and Soil Amended With Rock Phosphate

The effect of *Ochrobactrum* sp. SSR on wheat variety Faisalabad-2008 was evaluated in soil either amended or not with RP under controlled conditions. Increases in shoot length, root length, plant fresh weight and dry weight were recorded in plants with SSR and soil amended with RP as compared to the un-inoculated control. These changes were significant in the presence and absence of RP (Figures 2A,B; Table 2). The root morphological traits, i.e., root length, root volume, root surface area, root projection area, root tips and number of forks were also enhanced in inoculated plants (Figure 2C; Supplementary Table S3). Furthermore, we also observed increases in plant P (1.2–2.2%), available soil P (3.8–4.7 μg g^−1^) and alkaline phosphatase activity (5.6–6.2 μmoles g^−1^ of soil h^−1^) upon the treatment with the PSB (Table 2).

**Table 2:** Evaluation of PSB on wheat grown in soil amended with unavailable form of P under controlled conditions.

**Treatments**	**Fresh weight (g plant^**−1**^)**	**Dry weight (g plant^**−1**^)**	**Shoot length (cm)**	**Root Length (cm)**	**Plant P(%)^a^**	**Soil available P^b^**	**Phosphatase activity^c^**
Inoculated with RP	2.25 ± 0.11 A	1.12 ± 0.06 A	26.67 ± 1.33 A	11.71 ± 0.59 A	2.25 ± 0.16 A	4.70 ± 0.23 A	6.24 ± 0.31 A
Control with RP	1.06 ± 0.05 C	0.55 ± 0.03 BC	21.13 ± 1.06 BC	10.28 ± 0.51 BC	0.94 ± 0.07 C	3.12 ± 0.16 C	3.03 ± 0.15 C
Inoculated without RP	1.31 ± 0.07 B	0.65 ± 0.03 B	22.92 ± 1.15 B	11.05 ± 0.55 AB	1.19 ± 0.06 B	3.81 ± 0.19 B	5.65 ± 0.26 B
Control without RP	0.95 ± 0.05 C	0.48 ± 0.02 C	19.66 ± 0.98 C	9.85 ± 0.49 C	0.58 ± 0.03 D	2.33 ± 0.15 D	2.66 ± 0.15 D

*Evaluation of PSB on wheat growth, in soil amended with unavailable form of P under controlled conditions. The data were collected 30 DAS and shown as averages ± standard deviation of six replicates*.

a *Plant P Content is given in % of the total plant weight*,

b *Soil available P is represented by μg/gram of soil weight*.

c *Soil phosphatase activity is presented in μmoles/g of soil/h*.

*The mean values followed by different letters are significantly different at p > 0.05 according to LSD*.

### Shelf Life Study of Bioformulations

**Figure 3:**
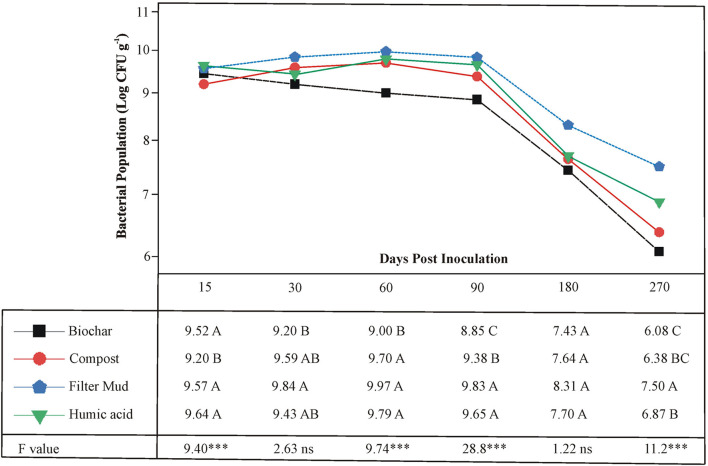
The shelf life of bioformulations to assess the survival of inoculated PSB in different carrier materials. Each point represents decimal logarithmic of viable cells g^−1^ carrier material and it is the mean value of three replicas. The ANOVA has been performed within each sampling date, thus within each column in data table, and the F and significance values (***p ≤ 0.001, ns not significant) are provided; the values followed by the same letter, within each column, are not significantly different at *p* < 0.05 in the LSD test.

The survival of the three PSB, *Ochrobactrum* sp. SSR, *Enterobacter* spp. ZW32, and ZW9, was evaluated in bioformulations that were based on four carrier materials. These included BC, CM, FM, and HA. The PSB were less abundant in CM than other carrier materials at 15 DPI (Figure 3). All carriers maintained the inoculated PSB up to 180 DPI. However, after the course of 270 days, the FM showed significantly higher levels of the PSB than other carrier materials.

**Figure 4:**
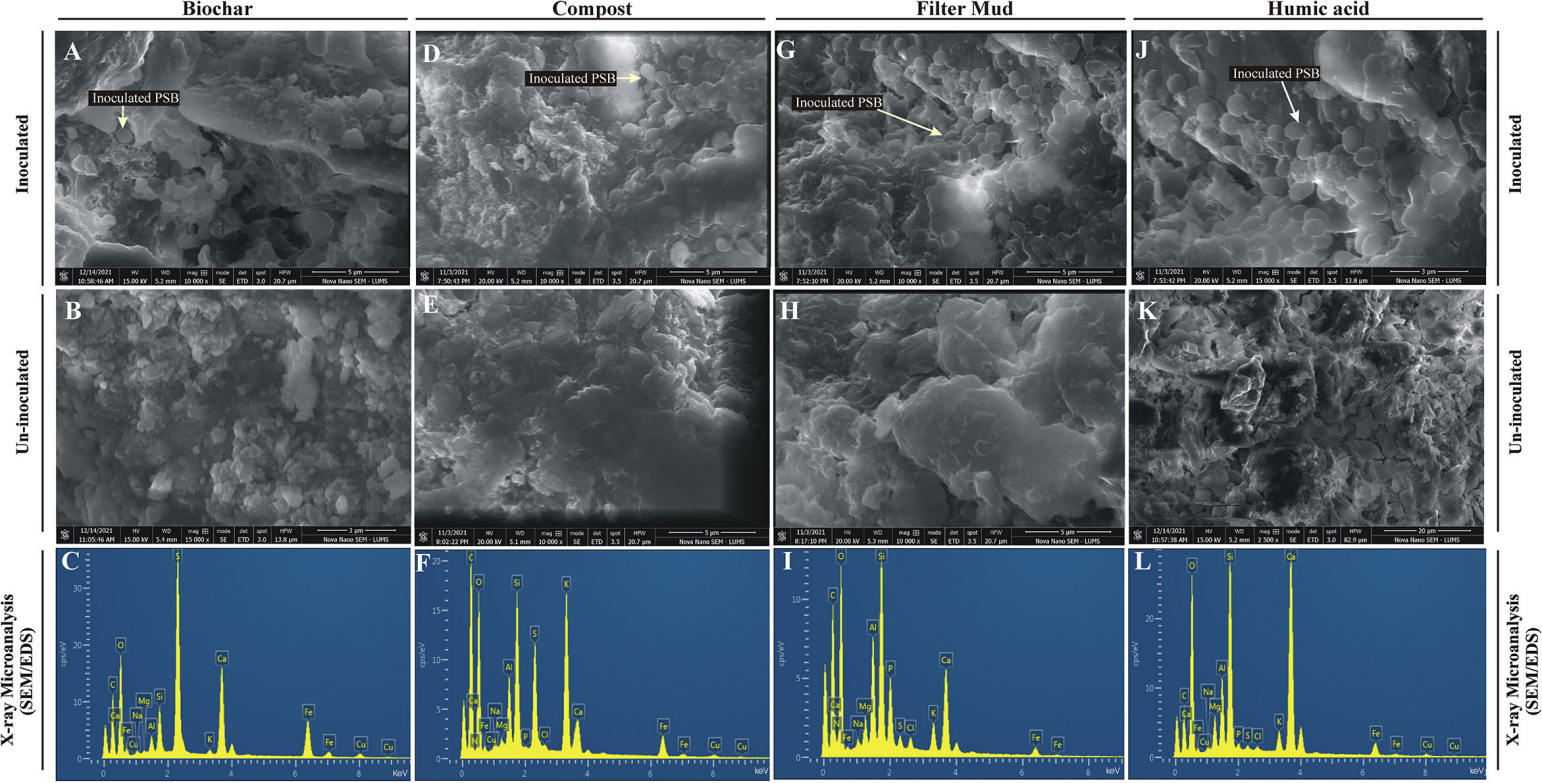
Field emission scanning electron microscopic analysis and energy dispersive X-ray spectroscopy of bioformulations. FESEM analysis of inoculated Biochar **(A)**, compost **(D)**, filter mud **(G)** and Humic acid **(J)** to visualize PSB and composition/ structure of carrier materials compared to non-inoculated filter mud **(B)**, compost **(E)**, Biochar **(H)** and Humic acid **(K)**. The chemical elements microanalysis of the filter mud **(C)**, compost **(F)**, Biochar **(I)** and Humic acid **(L)** is analyzed through EDS.

**Figure 5:**
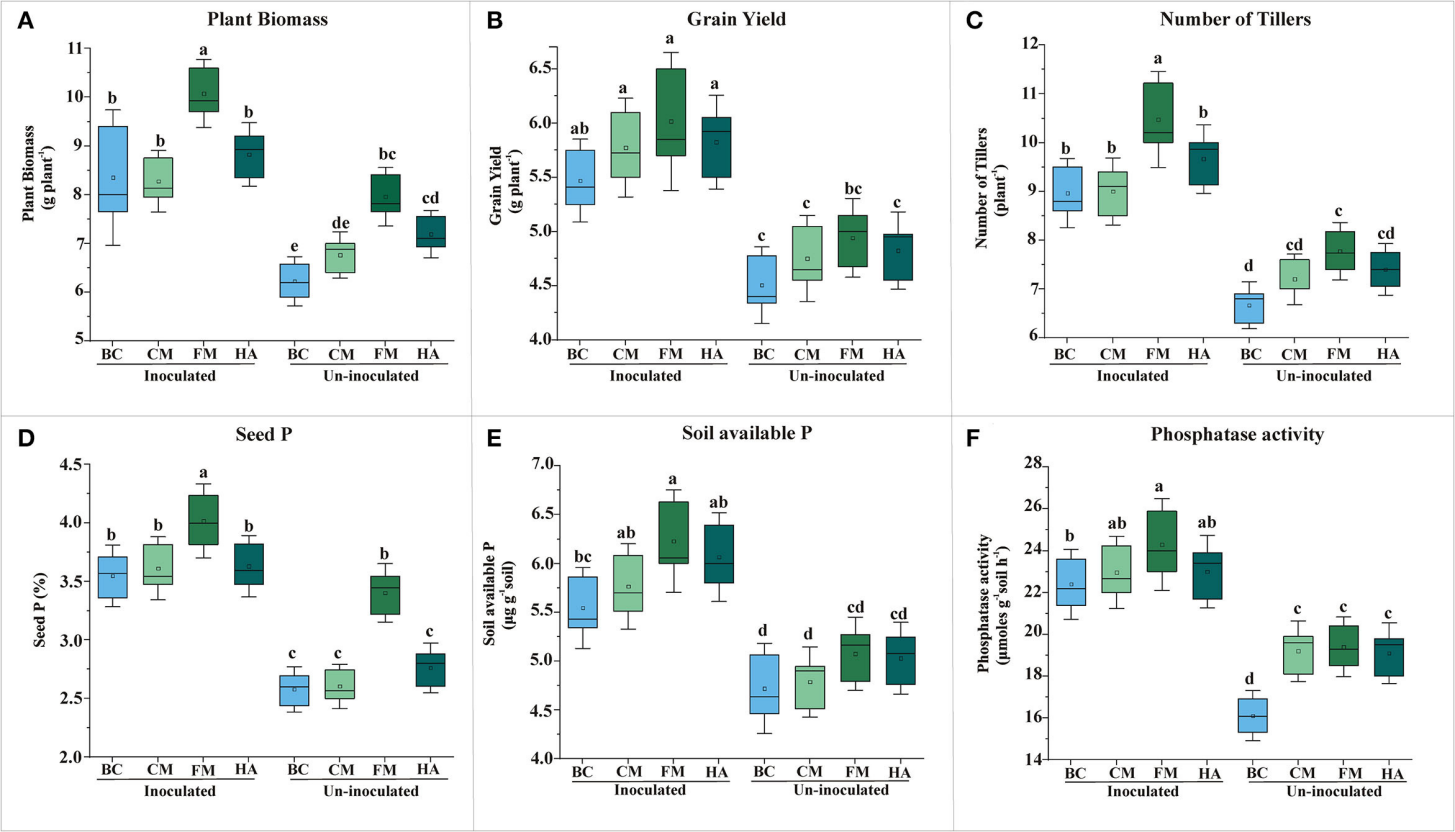
Evaluation of PSB bioformulations for plant yield and soil P contents of wheat grown soil compared to un-inoculated controls. The effect of bioformulation on plant biomass **(A)**, grain yield **(B)**, number of tillers **(C)**, seed P **(D)**, available P of soil **(E)**, and phosphatase activity **(F)**. The mean values are an average of six biological replicates organized in completely randomized design. The mean values followed by different letters are significantly different at *p* = 0.05. BC, Biochar; CM, Compost; FM, Filter mud; HA, Humic acid.

The inoculated PSB bacteria and the morphology of the bioformulations were investigated by FESEM. The PSB were detected in all four inoculated carrier materials (Figures 4A,D,G,J). Energy-dispersive X-ray spectroscopy was used to analyze the elemental composition of the surfaces of the bioformulations (Figures 4C,F,I,L). The data of SEM/EDS, CNHS, and biochemical analysis validates the presence of essential elements, i.e., N, K, C, and O in all tested carrier materials (Figure 4; Supplementary Table S4). However, it is also indicated that FM has more P than HA and CM. Only very low amounts of heavy metals Cu, Fe, and Cd were detected in the tested carrier materials (Figure 5).

### *In-vivo* Evaluation of Bio-Formulations in Earthen Pots Under Net House Conditions

All bioformulations enhanced the wheat plant growth in the pot experiment under net-house conditions up to variable extent (Figure 5). However, FM-based bioformulation showed significant increase in grain yield as compared to respective control, i.e., un-inoculated carrier material (Figure 5B). Seed P increased significantly in FM-based bioformulated plants compared to un-inoculated FM (Figure 5D; Supplementary Table S5), with concomitantly increased soil available P (6.23 μg g^−1^ soil) and alkaline phosphatase activity (24 μmoles g^−1^ of soil h^−1^) (Figures 5E,F; Supplementary Table S6).

### The Performance of the Filter Mud Bioformulation in the Field

Based on the persistence of inoculum in FM, and the highly promising plant growth promoting effects of the PSB with FM in the earthen pot experiment, FM-based bioformulation was tested under field conditions. The FM-based PSB bioformulation enhanced not only wheat grain yield and plant biomass, but also seed P, soil available P, and soil alkaline phosphatase activity (Table 3).

**Table 3:** Effect of Filter mud bioformulation and PSB consortium on plant growth and yield of wheat in microplots and field conditions.

**Microplot trial**	**Treatments**	**No. of tillers (tillers plant^**−1**^)**	**Plant height (cm)**	**Plant biomass (kg plot^**−1**^)**	**Grain yield (kg plot^**−1**^)**	**Seed P content (%)**	**Soil available P (μg g^**−1**^ soil)**	**Phosphatase activity(μmoles g^**−1**^ soil h^**−1**^)**
	Inoculated (80%)	13.33 ± 0.76 A	106.67 ± 5.03 A	2.36 ± 0.18 A	0.93 ± 0.04 A	4.15 ± 0.30 A	6.06 ± 0.28 A	26.63 ± 1.19 A
	Un-inoculated (80%)	8.53 ± 0.43 C	98.33 ± 5.03 A	1.93 ± 0.11 B	0.83 ± 0.04 B	3.36 ± 0.14 B	3.73 ± 0.27 C	19.33 ± 0.85 B
	Un-inoculated (100%)	10.07 ± 0.60 B	104.33 ± 5.51 A	2.11 ± 0.17 AB	0.87 ± 0.03 AB	3.68 ± 0.16 B	5.73 ± 0.31 B	24.73 ± 1.04 A
**Field trial**	**Treatments**	**No. of tillers (tillers m** ^**−2**^ **)**	**Plant height (cm)**	**Plant biomass (kg ha** ^**−1**^ **)**	**Grain yield (kg ha** ^**−1**^ **)**	**Seed P content (%)**	**Soil available P (μg g**^**−1**^ **soil)**	**Phosphatase activity (μmoles g**^**−1**^ **soil h**^**−1**^**)**
	Inoculated (80%)	468 ± 27 A	106 ± 5.31 A	14,667 ± 733 A	4,805 ± 224 A	4.60 ± 0.32 A	7.34 ± 0.34 A	26.67 ± 1.61 A
	Un-inoculated (80%)	369 ± 20 B	104 ± 5.03 A	12,317 ± 615 B	4,127 ± 220 B	3.30 ± 0.20 B	4.67 ± 0.20 C	19.67 ± 0.97 B
	Un-inoculated (100%)	389 ± 17 B	105 ± 5.22 A	13,167 ± 658AB	4,556 ± 210 AB	4.23 ± 0.23 A	5.91 ± 0.29 B	21.33 ± 1.12 B

*Wheat grown in microplots under net house and field conditions during winter wheat season 2018–2019. The FM bioformulation Inoculated and respective un-inoculated control were supplemented with 20% reduced application of DAP. Seeds were pelleted with FM bioformulation containing PSB consortium (1 × 10^9^ CFU ml^−1^). The mean values are an average of three biological replicates arranged in completely randomized design. The mean values followed by different letters are significantly different at p > 0.05 according to LSD. Area of each microplot was 2.25 m^2^ with a total number of 54 plants grown in each microplot. Each treatment of field trial has plot size of 6 m × 6 m and seed rate was @123.5 kg ha^−1^*.

**Figure 6:**
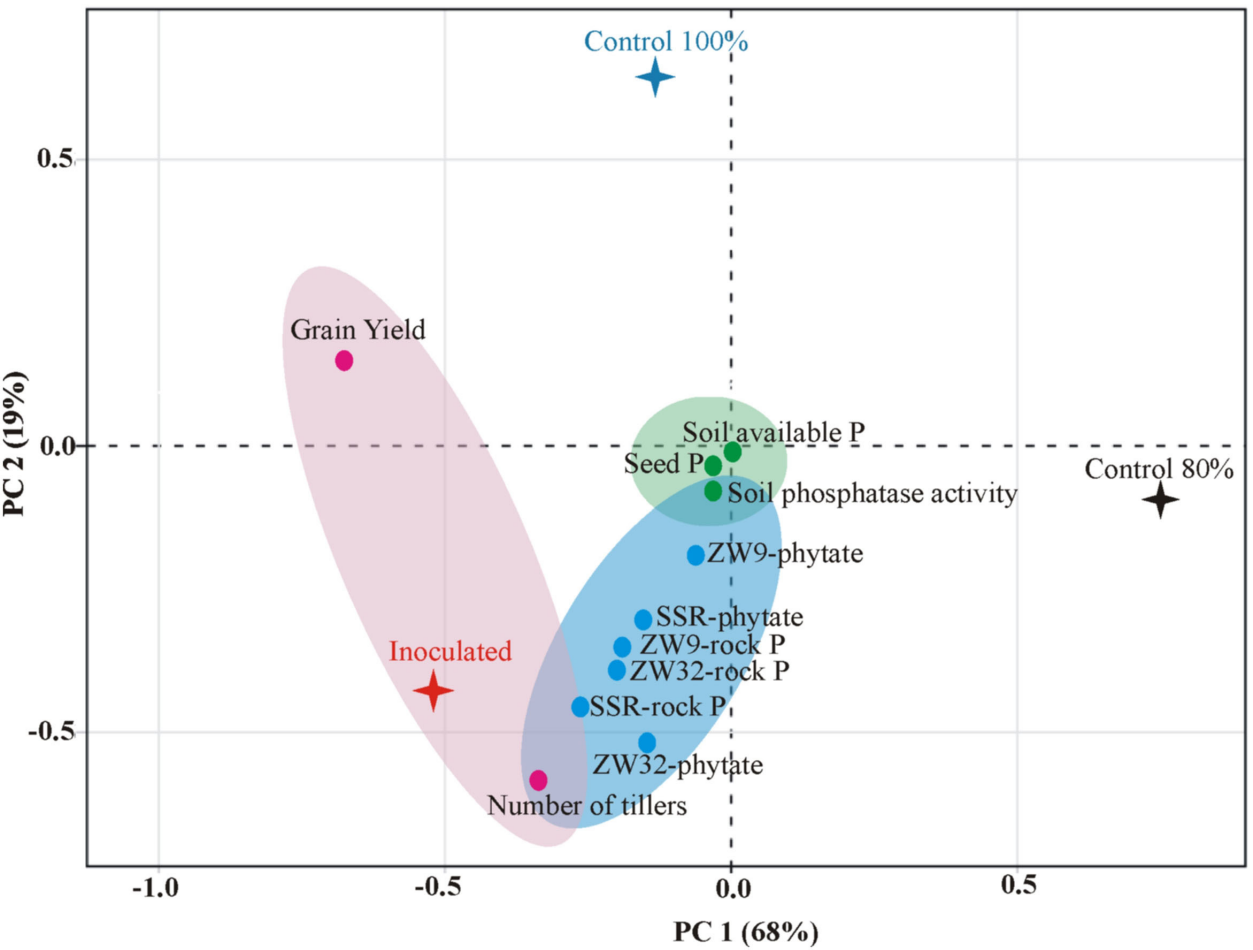
Principal component analysis depicting the response of PSB inoculation to wheat yield parameters, plant, and soil P content. The components elucidate a total variance of 87%. The points represent mean values of each combination of seed P, wheat yield parameters (grain yield and number of tillers), soil P contents (available P and phosphatase activity), and the treatments (PSB inoculation and un-inoculated controls).

A positive correlation was found between P-solubilization by SSR, ZW32, and ZW9 in presence of different insoluble P sources, and soil available P, soil phosphatase activity, seed P content, and grain yield of field grown inoculated wheat variety Faisalabad-2008 with 20% reduced DAP fertilization application (Figure 6). The PCA plot revealed correlation between different parameters, where the two principal components contributed up to 87% toward variance on *x*-axis (PC1 = 68%) and *y*-axis (PC2 = 19%). The plants inoculated with *Ochrobactrum* sp. SSR had significant (positive) impact on the grain yield, plant tillers, soil available P, soil phosphatase activity, seed P content of the wheat plants. No parameter was found to have negative effects by inoculation with the potential PSB.

### Detection of Inoculated PSB

**Figure 7:**
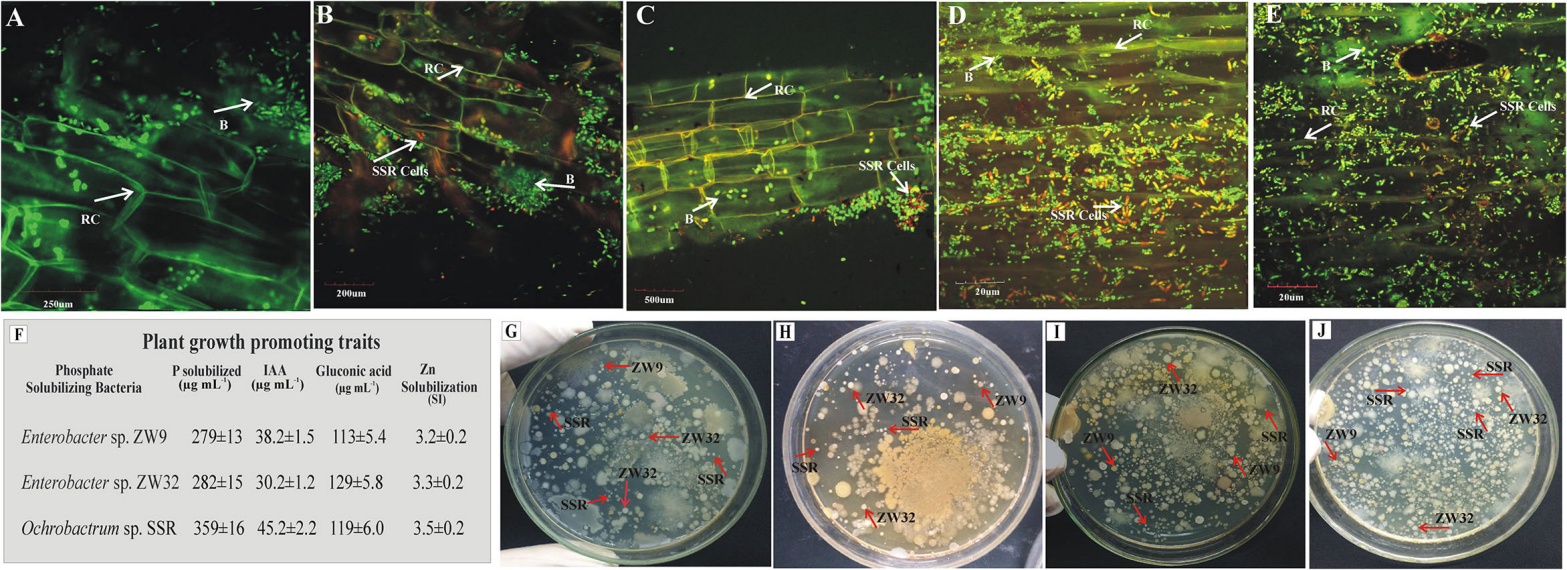
Re-isolation and detection of inoculated P-solubilizing bacteria. The CLSM of wheat roots at 35 days post inoculation of P-solubilizing bacteria in the pot experiment under net house conditions. The FLUOS-labeled oligonucleotide probes showed green fluorescent signals for total bacterial population and Cy3-labeled oligonucleotide probes showed red fluorescent signals for inoculated *Ochrobactrum* sp. SSR. **(A)** Control treatments without inoculation and from the PSB inoculated pots with carrier materials **(B)** Biochar, **(C)** CM, **(D)** FM, and **(E)** HA after hybridizing with fluorescently labeled probes. The plant growth promoting traits of re-isolated PSB strains for their confirmation in comparison to pure PSB **(F)**. The plates depicting the re-isolated colonies of SSR, ZW32, and ZW9 from BC **(G)**, CM **(H)**, FM **(I)** and HA **(J)** in inoculated treatments. *Enterobacter* spp. ZW9, ZW32, *Ochrobactrum* sp. SSR. B, bacteria; RC, root cells.

The presence of inoculated PSB in wheat rhizosphere soil from earthen pot experiment was confirmed by plating and counting the bacteria and FISH at 35 days after seeding (Figure 7). The root colonization was studied by FISH–CLSM of inoculated wheat roots. The FLUOS-labeled green probe EUB338 showed the total bacterial population (Figure 7). The Cy3-labeled ALF1b probe predominantly detects the presence of *Ochrobactrum* sp. SSR population in the PSB inoculated treatments as indicated by red cells (Figures 7B–E). Of note, the highest density of the red cells was observed in FM carrier material (Figure 7D). The re-isolated colonies of PSB, i.e., *Ochrobactrum* sp. SSR*, Enterobacter* spp. ZW32, and ZW9 were identified based on their morphological characteristics and plant growth promoting attributes, i.e., phosphate solubilization (279–359 μg ml^−1^), gluconic acid production (113–129 μg ml^−1^), and indole acetic acid production (30–45 μg ml^−1^).

## Discussion

The development and use of the microbial inoculants has elicited great interest in the recent years since it presents a sustainable alternative for the application of chemicals fertilizes (Romano et al., 2020). However, the biofertilizer products still face major challenges, associated with the limited shelf life and the persistence of inoculant in different environments, and this limits their use in agriculture (Basu et al., 2021). To tackle this problem, we constructed a bioformulation that entails three plant growth promoting bacteria carried by FM. Our results suggest that the designed bioformulation has a long shelf life, is persistent in soil, and effective for both greenhouse and field applications.

The applied bacterial strains solubilized RP and phytate. Previously these bacteria were reported to solubilize P from tricalcium phosphate (Yahya et al., 2021). Our data supports the view that the utilization of multiple P sources by bacteria assures that they release P in different substrates and environments, and exhibit a strong potential for applications (Wan et al., 2020). We suggest that the successive analysis of TCP, RP and phytate solubilization by potentially novel plant beneficial bacteria provides a useful approach to obtain PSB that release P effectively in different soils.

To determine the genetic features of SSR that may contribute to the higher P solubilization, *pqqC* gene related to PQQ biosynthesis was amplified and analyzed phylogenetically. To this end new primers were designed that specifically amplify *pqqC* gene of *Ochrobactrum* genus. The *pqqC* encodes pyrroloquinoline quinone biosynthesis protein C, a well characterized enzyme that catalyze the final step of PQQ biosynthesis pathway. It is involved in oxidation and cyclization of 3*a*-(2-amino-2-carboxy-ethyl)-4 and 5-dioxo hexahydoquinoline dicarboxylic acid in the final step of PQQ biosynthesis (Meyer et al., 2011). In our previous studies, we revealed *gcd* (accession numbers MK883706), *pho* (accession numbers MK883704; Rasul et al., 2021), and *pqqE* genes (accession numbers MT897167; Yahya et al., 2021) containing *Ochrobactrum* sp. SSR is multifaceted PSB. The *pqqC* and *pqqE* genes of strain SSR were amplified and known as most essential genes of PQQ operon. The biosynthesis of PQQ is only accomplished when the complex of gene products, i.e., pqq A, B, D, and F gets attached to pqqC at the C-terminal and with pqqE at the N-terminal (Bhanja et al., 2021). The amplification of these essential genes is the significant contribution and can provide novel insights into understanding the PQQ biosynthesis pathway in *Ochrobactrum* sp.

New primers for qPCR were designed and reported for the first time as marker genes to directly link the gene expression with phosphate-solubilizing activity. These primers were successfully applied for real time quantitative analysis of functional genes, i.e., *pqqC, pho* and *gcd* in inoculated soils of a pot experiment. We observed higher soil available P and soil phosphatase activities with concomitant increased expression of *pqqC* and *pho* genes related to SSR in RP amended soil as compared to inoculated soil with available P. This significant increase in soil available P corresponds to high rock P-solubilizing potential of *Ochrobactrum* sp. SSR *in vitro*. The SSR performed profoundly even in the absence of un-available P source but more efficiently in the presence of un-available P rock-phosphate as validated by higher expression level of marker genes, i.e., *pqqC* and *pho* relative to the *gcd* expression level. The developed qPCR primers are useful molecular tools to quantitatively trace functional gene expression and abundance of SSR that can be linked to its P-solubilizing activity in the wheat rhizosphere. Our results are in line with globally coordinated nutrient addition experiments carried out in the UK, the USA, and South Africa by Widdig et al. (2019), which described that the P-solubilizing microbes can work more effectively in nutrient deprived soils. This leads to an increase in relative-abundance of PSB in plant rhizosphere. Similarly, a case study of rhizosphere soil from temperate beech forest revealed P-solubilizing efficacy of PSB get lowered in nutrient rich soils than soils with low nutrient availability (Nicolitch et al., 2016). Moreover, another study demonstrated that application of PSB in the soil significantly increased the soil available P and soil phosphatase activity in semi-arid soils with concomitant increased expression of *gdh* and *pqqC* genes in a microcosmic experiment (Saadouli et al., 2021).

The effects of PSB inoculation were not limited to phosphate solubilization only, but also extended to multiple root morphological traits, thus improving acquisition of P (Table 2). Inoculated wheat plants showed significant improvement in both above ground plant traits (i.e., plant P and shoot length) and belowground plant traits (i.e., root length, soil available P, and phosphates activity). This finding is consistent with the recent study by Elhaissoufi et al. (2020), reported improved above and below ground parameters of PSB-inoculated wheat plants. The root morphological traits indicated root proliferation that significantly correlated with root/shoot P contents. This roots foraging and proliferation could lead to a greater P uptake.

After the confirmation of genetic potential of SSR to solubilize recalcitrant-P, the present work is focused on designing the optimal inoculant formulation based on this elite strain SSR along with two other well-characterized and compatible PSB strains *Enterobacter* spp. ZW32 and ZW9 to develop consortium for wheat to be applied under field conditions. Application of consortium can improve plant growth and increase the crop yield as compared to application of single bacterium (Backer, 2018; Mpanga et al., 2019). Although an elite PSB strains with consistent performance under field conditions is essential for successful inoculant development, the non-biological components are still the key bottlenecks in commercial development of the inoculants (Pastor-Bueis et al., 2019; Mendoza-Suárez et al., 2021). Four different carrier materials, i.e., BC; CM; FM, and HA were used to design bioformulations with PSB-consortium based on autochthonous strain *Ochrobactrum* sp. SSR. Two other PSB strains used in consortium were well characterized *Enterobacter* spp. DSM 109592 and DSM 109593.

The elemental analysis of all carrier materials was performed with SEM accompanied with EDS. The data of SEM/EDS (Figure 4), CNHS, and biochemical analysis validates the presence of essential elements, i.e., N, K, C, and O in the tested carrier materials (Supplementary Table S4). However, P content of FM was higher than HA and CM. Non-significant amount of heavy metals Cu, Fe, and Cd were detected in all tested carrier materials. The essential heavy metals such as Cu, Fe, Mn, and Zn are required for various biochemical and physiological processes of plant (Yan et al., 2020), but they may become toxic when present in excess. Therefore, the carrier materials with low amount of essential heavy metals are the potential candidates for the development of successful bioformulation.

The shelf-life assessment of well-characterized biostimulants revealed that all carriers maintained adequate survival of PSB to a variable extent up to 180 days period. The FM showed significantly higher bacterial load as compared to rest of the tested carrier materials predominantly up to 270 DPI (Figure 3). The persistence of inoculated PSB in carrier materials was further validated by FESEM. This indicates that FM-based carrier material provided more suitable microenvironment for inoculated PSB and maintained longer shelf life. This is an essential attribute of carrier materials for maintenance of microbial-viability without any special need of storage facility (Soumare et al., 2020). Besides, very few studies have focused on selection and development of carrier materials and their effect on bioinoculum as most of studies emphasized on the performance of microbial inoculants (Koskey et al., 2021).

After designing of potential biostimulants with adequate shelf life, the next step was to evaluate the inoculants under field conditions. The FM-based bioformulation showed a pronounced effect on wheat growth as indicated by significant improved grain yield (16%) and seed P content (15%) with concomitant inceresed in soil available P (18%) under net house conditions (Figure 5). Under the field conditions, significant increase in grain yield (5%), plant biomass (10%), soil available P (7%), and soil phosphatase activity (27 μmol g^−1^ soil h^−1^) were observed in FM-based bioformulation compared to un-inoculated 80 and 100% controls (Table 3). The results are in line with some previous studies that shows the enhanced rice growth (Tahir et al., 2013) and wheat grain yield (Suleman et al., 2018) under agro–ecological conditions when seed pelleted with FM and PSB. Therefore, the FM can be adopted as a potent carrier material for the PSB aimed at improved soil nutrient availability, enhanced wheat grain yield, and decipher a key role as green alternative for imminent agricultural sustainability. Multiple traits of the PSB are directly involved in the bioavailability and P uptake by the plants. The PSB-enhanced nutrient-sensing capacity of the plants, which has already been made available by the secretion of bacterial organic acids (Chungopast et al., 2021; Yahya et al., 2021). The PSB modulates the morphology of root architecture and alleviates the P-stress by making P available in the soil and stimulating the plants to uptake P. This tripartite system could lead to improved crop vigor and yield.

The principal component analysis indicates a positive correlation between P solubilization by SSR, ZW32, and ZW9 in presence of different insoluble phosphate sources, and soil available-P, soil phosphatase activity, seed P content and grain yield of field grown inoculated wheat variety Faisalabad-2008 with 20% reduced application of DAP fertilizer (Figure 6). The agronomic performance of the potential inoculant tested under field trial, appraise the superiority of the inoculant. The various studies demonstrate that P-solubilizing microbes showed best effect with reduced application of DAP fertilizers (Suleman et al., 2018; Rosa et al., 2020; Rasul et al., 2021; Yahya et al., 2021).

The survival of inoculated PSB strains in wheat-rhizosphere was validated by viable count and FISH-CLSM, this shows that the inoculated PSB were rhizosphere-competent phosphobacteria. Furthermore, the plant growth promoting attributes of re-isolated PSB were compared to their pure cultures, indicates the persistence of inoculated PSB (Figure 7). The persistent colonization of PGPR in the rhizosphere is an indication that bacteria can carry out their functions (Hakim et al., 2021; Lopes et al., 2021) and form an association with local microbial community (Santoyo et al., 2021).

The ecological approach developed in the present study enabled us the development of biostimulators that can act as a soil conditioner in agriculture. These potential formulations can be a substitute to chemical fertilizers to optimize the use of natural phosphate in the world and improve resilience and yield of wheat crop. The deployment of such biostimulants containing multifaceted autochthonous *Ochrobactrum* sp. SSR and *Enterobacter* spp. ZW32 and ZW9 can offer a potential approach to mitigate P-induced stress in wheat plants. *Ochrobactrum* sp. SSR exhibit pronounced potential in terms of modification of root morphology/architecture and increased real-time PS related genes expression to aid in transformation of insoluble P compounds into available forms for plant uptake, stimulate plant growth, and increase the crop yield.

## Conclusions

This study provides a solid foundation to design well-characterized biostimulant formulation that deployed the multifaceted PSB and organic amendments based on agro-industrial by-products to improve the wheat growth and yield under P-deficient conditions. This is the first comprehensive research work that reports amplification of PS-related genes, i.e., *gcd, pqqC, pqqE*, and *pho* and real time expression of these genes by autochthonous *Ochrobactrum* sp. SSR, to release P from recalcitrant P forms. The successful biostimulant developed in this study is not only well-characterized but also extended the shelf life of highly efficient rhizosphere-competent P-solubilizers that ultimately led to sustainable wheat production for global food security.

## Data Availability Statement

The datasets presented in this study can be found in online repositories. The names of the repository/repositories and accession number(s) can be found in the article.

## Author Contributions

MY analyzed the data, wrote the manuscript, and executed statistical analysis. SY and MT performed data analysis and review the manuscript. MTT, TR, MR, and MY designed the primers. YS, IA, and MY performed the expression analysis. ZS, MT, and MS performed elemental analysis of carrier materials. AI helped in FISH analysis and in biochemical analysis of soils. SY conceived and supervised the whole study and edited the manuscript. All authors contributed to the article and approved the submitted version.

## Conflict of Interest

The authors declare that the research was conducted in the absence of any commercial or financial relationships that could be construed as a potential conflict of interest.

## Publisher's Note

All claims expressed in this article are solely those of the authors and do not necessarily represent those of their affiliated organizations, or those of the publisher, the editors and the reviewers. Any product that may be evaluated in this article, or claim that may be made by its manufacturer, is not guaranteed or endorsed by the publisher.
