# Supplementary material for: Designing Synergistic Biostimulants Formulation Containing Autochthonous Phosphate-Solubilizing Bacteria for Sustainable Wheat Production
Source: Front Microbiol. 2022 May 3;13:889073. doi: 10.3389/fmicb.2022.889073 (PMC9111743; doi:10.3389/fmicb.2022.889073)
Supplement: Supplementary file 1 [file Table_1.DOCX]

**Table S1:** Quantification of P solubilized by PSB in liquid rock phosphate medium and phytate medium at different time intervals

There were six biological replicates and ± represents Standard Deviation. Means with significant difference (P*<*0.01) among treatments is represented by different letter. DPI: Days Post Inoculation.

*Quantification of P solubilized by *Ochrobactrum* sp. SSR has been reported in our preceding study (Rasul et al., 2021).

| **Phosphate solubilizing bacteria** | **Phosphate solubilized**  **(µg mL^-1^)** | | | | | | | | | |
| --- | --- | --- | --- | --- | --- | --- | --- | --- | --- | --- |
| **P source:** | **Rock Phosphate** | | | | | **Phytate** | | | | |
|  | 3 DPI | 5 DPI | 7 DPI | 10 DPI | 15 DPI | 3 DPI | 5 DPI | 7 DPI | 10 DPI | 15 DPI |
| *Enterobacter* sp. ZW9 | 40±2.37 | 57±2.85 | 77±3.53 | 79±4.10 | 90±4.51 | 92±4.51 | 85±4.43 | 78±3.93 | 46±2.34 | 13±0.65 |
| *Enterobacter* sp. ZW32 | 45±2.25 | 60±3.44 | 65±3.72 | 88±4.45 | 97±4.79 | 93±4.75 | 88±4.79 | 81±4.05 | 108±5.43 | 18±0.89 |
| *Ochrobactrum* sp. SSR* | 58±3.18 | 93±4.65 | 96±4.82 | 99±4.98 | 109±5.7 | 136±6.8 | 222±11.1 | 91±4.55 | 44±2.22 | 22±1.17 |

**Table S2.** Treatments for evaluation of bio‑formulations in earthen pots under net house conditions

| **Treatments** | **Discription** |
| --- | --- |
| T1 | Seeds pelleted with Inoculated Biochar |
| T2 | Seeds pelleted with Inoculated Compost |
| T3 | Seeds pelleted with Inoculated Humic acid |
| T4 | Seeds pelleted with Inoculated Filter Mud |
| T5 | Seeds pelleted with Un-inoculated Biochar |
| T6 | Seeds pelleted with Un-inoculated Compost |
| T7 | Seeds pelleted with Un-inoculated Humic acid |
| T8 | Seeds pelleted with Un-inoculated Filter Mud |

**Table S3.** Effect of phosphate solubilizing bacteria on root morphological traits of wheat plants grown in soil amended with unavailable form of P under controlled conditions

| **Treatments** | **Root Length (cm)** | **Root surface**  **area**  **(cm^2^)** | **Root**  **diameter**  **(mm)** | **Root volume**  **(cm^3^)** | **Area of**  **projection**  **(cm^2^)** | **Number of**  **root tips** | **Number of**  **Forks** | **No. of**  **Crossings** |
| --- | --- | --- | --- | --- | --- | --- | --- | --- |
|  |  |  |  |  |  |  |  |  |
| **Inoculated with RP** | 579±31.14 A | 56.96±3.98 A | 0.37±0.02 A | 0.428±0.020 A | 17.15±0.87 A | 812±36.69 A | 3476±158 A | 0.426±0.05 A |
| **Control with RP** | 444±21.38 C | 33.97±2.04 C | 0.34±0.02 AB | 0.287±0.016 B | 09.63±0.40 B | 421±28.58 C | 1962±108 C | 0.287±0.08 A |
| **Inoculated without RP** | 508±26.57 B | 48.48±2.16 B | 0.36±0.02 A | 0.401±0.018 A | 16.48±0.71 A | 562±37.00 B | 2951±147 B | 0.401±0.02 A |
| **Control without RP** | 392±16.69 D | 33.60±2.05 C | 0.321±0.01 B | 0.286±0.013 B | 08.88±0.47 B | 354±14.36 D | 1906±109 C | 0.286±0.05 A |

Evaluation of PSB in soil amended with unavailable form of P under controlled conditions. Data is an average of six replicates. Data was taken at 30 Days after sowing (DAS). ± represents Standard Deviation. Means with significant difference (P*<*0.05) among treatments is represented by different letter. PSB (1 x10^9^ CFU mL^-1^) was seed-inoculated before sowing.

**Table S4.** Bio-chemical properties of different carrier-based bio-formulations

| **Characteristics** | **Carrier materials** | | | |
| --- | --- | --- | --- | --- |
|  | **Biochar** | **Compost** | **Filter mud** | **Humic acid** |
| EC (dS m^-1^) | 3.44±0.18 | 3.05±0.20 | 3.00±0.16 | 3.44±0.18 |
| pH | 7.10±0.46 | 7.00±0.50 | 7.20±0.52 | 7.70±0.58 |
| Total P (%) | 1.03±0.08 | 0.82±0.14 | 2.60±0.08 | 0.45±0.08 |
| Total N (%) | 1.42±0.08 | 3.20±0.18 | 4.50±0.21 | 1.23±0.09 |
| Organic matter | 75±4.08 | 60±3.15 | 65±3.54 | 35±2.85 |

Bio-chemical properties of different carrier-based bioformulations. All values are an average of six biological replicates.

**Table S5.** Effect of different carrier-based bio-formulations and PSB consortium on wheat growth in a pot experiment under net house conditions

|  | Treatments | 35 DAS^1^ | | | |  |  | | 120 DAS^2^ | | | | |
| --- | --- | --- | --- | --- | --- | --- | --- | --- | --- | --- | --- | --- | --- |
|  |  |  | | | |  |  | |  | | | | |
|  |  | Plant Weight  (g plant^-1^) | Shoot Length (cm) | Root Length (cm) | Plant P content (%) | Viable (CFU/ g soil) | | No. of tillers (per plant) | | Plant Height (cm) | Plant Biomass (g/ plant) | Grain Yield (g/ plant) | Seed P content (%) |
| Inoculated | Biochar | 1.67±0.08 B | 32.22±1.61 B | 9.60±0.55 BC | 3.73±0.19 C | 8.84x10^7^ | | 8.97±0.47 B | | 76.51±3.84 A | 8.35±0.93 B | 5.50±0.24 AB | 3.55±0.18 B |
|  | Compost | 1.63±0.08 B | 33.00±1.65 B | 10.45±0.50 AB | 3.77±0.19 BC | 9.13x10^7^ | | 9.00±0.46 B | | 77.32±3.85 A | 8.28±0.42 B | 5.77±0.26 A | 3.61±0.18 B |
|  | Filter mud | 1.86±0.07 A | 36.00±1.95 A | 11.33±0.58 A | 4.63±0.32 A | 9.53x10^7^ | | 10.47±0.64 A | | 78.20±3.90 A | 10.08±0.47 A | 6.03±0.43 A | 4.02±0.21 A |
|  | Humic acid | 1.81±0.05 A | 34.67±1.73 AB | 10.67±0.58 AB | 4.08±0.20 B | 9.39x10^7^ | | 9.67±0.47 B | | 78.06±3.97 A | 8.83±0.43 B | 5.83±0.29 A | 3.63±0.18 B |
| Un-Inoculated | Biochar | 0.81±0.04 D | 22.67±1.13 D | 08.00±0.40 D | 2.00±0.10 F | 7.18x10^7^ | | 6.67±0.32 D | | 75.31±3.72 A | 6.23±0.34 E | 4.50±0.24 C | 2.58±0.13 C |
|  | Compost | 0.83±0.04 D | 24.23±1.23 D | 08.00±0.40 D | 2.33±0.11 E | 6.93x10^7^ | | 7.20±0.35 CD | | 75.55±3.88 A | 6.76±0.32 DE | 4.80±0.26 C | 2.60±0.13 C |
|  | Filter mud | 0.93±0.06 C | 28.33±1.42 C | 09.33±0.46 C | 3.47±0.16 CD | 7.80x10^7^ | | 7.77±0.39 C | | 76.46±3.78 A | 7.96±0.40 BC | 4.97±0.24 BC | 3.40±0.17 B |
|  | Humic acid | 0.92±0.05 C | 28.00±1.40 C | 08.63±0.43 CD | 3.32±0.17 D | 7.73x10^7^ | | 7.40±0.35 CD | | 76.09±3.57 A | 7.19±0.32 CD | 4.87±0.24 C | 2.76±0.14 C |

^1, 2^ Effect of carrier-based bioformulations and PSB consortium on various wheat growth parameters at 35 days after sowing (DAS) and at 120 DAS in a pot experiment. Inoculated and Un-inoculated controls supplemented with 80% of the recommended dose of DAP fertilizer. PSB consortium (1 ×10^9^ CFU mL^-1^) were seed-inoculated before sowing. Means are the average of six replicates arranged in CRD. Means followed by the same letter differ non-significantly at p = 0.05 according to LSD. ± represents the standard deviations (SD). Plants were grown in earthen pots (Diameter: 12 inch, Height: 14 inch) containing 12 kg soil. Six plants were grown per pot.

**Table S6.** Effect of different carrier-based bio-formulations and PSB consortium on soil P and phosphatase activity in a pot experiment under net house conditions

|  | **Treatments** | **35 DAS^1^** | | **120 DAS^2^** | |
| --- | --- | --- | --- | --- | --- |
|  |  | Soil Available P  (μg g^-1^ soil) | Phosphatase Activity  (μmoles g^-1^ soil hr^-1^) | Soil Available P  (μg g^-1^ soil) | Phosphatase Activity  (μmoles g^-1^ soil hr^-1^) |
| **Inoculated** | Biochar | 5.29±0.26 B | 10.80±0.54 A | 5.54±0.28 BC | 22.40±1.12 B |
|  | Compost | 5.49±0.27 B | 10.60±0.53 A | 5.76±0.29 AB | 22.97±1.15 AB |
|  | Filter mud | 6.30±0.31 A | 11.30±0.43 A | 6.23±0.35 A | 24.10±1.21 A |
|  | Humic acid | 5.58±0.28 B | 11.05±0.55 A | 6.06±0.30 AB | 23.00±1.15 AB |
| **Un-Inoculated** | Biochar | 4.54±0.26 C | 7.73±0.39 C | 4.72±0.31 D | 16.10±0.80 D |
|  | Compost | 4.63±0.26 C | 7.87±0.40 C | 4.78±0.24 D | 19.20±0.96 C |
|  | Filter mud | 4.78±0.22 C | 9.60±0.48 B | 5.07±0.25 CD | 19.40±0.95 C |
|  | Humic acid | 4.71±0.26 C | 7.84±0.36 C | 5.03±0.25 CD | 19.10±0.96 C |

^1, 2^ Effect of carrier-based bioformulations and PSB consortium on soil P and phosphatase activity at 35 days after sowing (DAS) and at 120 DAS in a pot experiment. Inoculated and Un-inoculated controls supplemented with 80% of the recommended dose of DAP fertilizer. PSB consortium (1 ×10^9^ CFU mL^-1^) were seed-inoculated before sowing. Means are the average of six replicates arranged in CRD. Means followed by the same letter differ non-significantly at p = 0.05 according to LSD. ± represents the standard deviations (SD). Plants were grown in earthen pots (Diameter: 12 inch, Height: 14 inch) containing 12 kg soil. Six plants were grown per pot
